# Molecular docking analysis of lupeol with different cancer targets

**DOI:** 10.6026/97320630018134

**Published:** 2022-03-31

**Authors:** Mahalakshmi Gunasekaran, Ravali Ravi, Kavimani Subramanian

**Affiliations:** 1 Department of Pharmacology, College of Pharmacy, Mother Theresa Post Graduate and Research Institute of Health Science, Pondicherry University, Puducherry-605006, India

**Keywords:** Molecular docking, lupeol, cancer targets

## Abstract

Lupeol is one of the secondary metabolite (triterpenoid) present in many medicinally effective plants. It has numerous biological and pharmacological actions. Lupeol is found to have effective herbs and has immense biological activity against several diseases including its cytotoxic effect on cancer cells. In recent drug designing, molecular study of analysis is usually used for understanding the target and the ligand interaction. Therefore, it is of interest to document the molecular docking analysis data of lupeol with different cancer targets such as Caspase- 3, BCL-2, Topoisomerase, PTK, mTOR, H-Ras, PI3K, and AKT. These molecular docking studies were carried out by using AutoDock tools 4.2 version software. Molecular docking analyses of lupeol with target protein were found to have good dock score and minimum inhibition constant. BCL-2, Topoisomerase, PTK, mTOR and PI3Kdocking studies showed the best binding energy inhibition constant and ligand efficiency. The in-silico molecular docking analysis showed that the lupeol having relatively good docking energy, affinity and efficiency towards the active macromolecule, thus it may be considered as good inhibitor of proliferating cancer cells. By this knowledge of docking results, the lupeol can be used as promising drug for anticancer activity.

## Background:

Lupeol is a penta cyclic tri terpenoid, present in most of the effective herbs and exhibits an immense biological activity against human ailnments [1, 2]. Lupeol has cytostatic effects on cancer cells through modulation of expression of IL-2, IL4, IL5, ILβ, proteases, α-glucosidase, cFLIP, and NFκB [2-5].Also significantly induces cell deaths through altering the expression levels of BCL-2, BAX, caspases, and PI3K-AKT-mTOR signaling pathway in cancer cells [1, 5-7]. It modulates the molecules such as Cyclins, CDKs, P53, P21, PCNA, cdc25C, and plk1 which were involved in cell cycle regulation in different cancer types [7, 8].Cancer cells have a characteristic metabolism, mostly caused by alterations in signal transduction networks rather than mutations in metabolic enzymes [9].To develop targeted therapies, identification of the genetic changes that help a tumor to grow and change is necessary. A potential target would be a protein that is present only in cancer cells but not healthy cells. This can be caused by a mutation. Targeted therapy in cancer inhibits the signaling pathway of the targets which carry information regarding enhanced cell growth. Many research works on the anticancer activity of lupeol has been reported. Not many reports have been published on the in-silico docking approach. So an attempt has been made to study the clear mechanism of action with the aid of in-silico approaches. Few target proteins like Caspase-3, BCL-2, Topoisomerase, Protein tyrosine kinases (PTK), Phosphatidylinositol-3-kinase (PI3k), and Mammalian or Mechanistic target of rapamycin (mTOR), AKT, H-ras. Caspase-3 is an endoprotease enzyme that coordinates the destruction cellular structures like DNA fragmentation and degradation of cytoskeletal proteins. Caspases are essential in the dismantling processes of the cell and the formation of apoptotic bodies [10-12].The deregulation of caspase-3 leads to cancer. BCL-2 is B-cell lymphoma 2, encoded in humans by the BCL-2 gene that regulates apoptosis. An unbalanced state between pro- versus anti-apoptotic BCL-2 proteins can act as a barrier to apoptosis and facilitate cancer development [13, 14]. Topoisomerases are one of the most important cancer chemotherapy targets [15, 16]. These enzymes play a crucial role for cell function and perform a wide range of functions like maintenance of DNA topology in DNA replication, and transcription. Protein tyrosine kinase (PTK) is one of the major signaling enzymes in the process of cell signal transduction that regulates cell growth and differentiation [17-19]. Aberration in this pathway leads to various forms of cancer [20]. Over 40 chromosomal translocations with of 12 different PTK deregulated signaling were associated with various hematologic malignancies [21]. Phosphoinositide 3-kinase (PI3K) and its subtypes regulate AKT signaling pathway with the help of numerous stimuli and kinases present in the cell which leads cellular growth and survival [22-24]. mTOR with other key components catalyzes the phosphorylation of multiple targets such as ribosomal protein S6 kinase β-1 (S6K1), eukaryotic translation initiation factor 4E binding protein 1 (4E-BP1), AKT, protein kinase C (PKC), and type-I insulin-like growth factor receptor (IGF-IR), and regulates protein synthesis, nutrients metabolism, growth factor signaling, cell growth, and migration. Thus deregulation of mTOR leads to tumor growth and metastasis [25-27]. The Akt (serine/threonine kinase) is a central node of many signaling pathways and it is often deregulated in most of the cancers [23,28]. The abnormal over expression or activation of Akt leads to increased cell proliferation and survival which is observed in many cancers including breast, ovarian, pancreatic, and lung cancers [29,30]. Ras mutant protein regulates tumor cell proliferation, apoptosis, metabolism and angiogenesis through downstream MAPK, PI3K and other signaling pathways [31]. All mammalian cells express 3 closely related Ras proteins, termed H-Ras, K-Ras, and N-Ras, that promote oncogenesis when they are mutationally activated at codon 12, 13, or 61 [32].

## Material and Methods:

Molecular modelling technique is very most important method for the investigation and reorganization of receptor protein and compound structure potentially without giving more exertion and investment in research work [31-35]. Structure prediction of target and the ligand is important for their interaction studies [36].

## Preparation of ligand:

Lupeol is chosen as a ligand. The structure of ligand is obtained from PubChem database. The structural information was collected by using PubChem. The structure of the molecule can be drawn with the help of marvin sketch software namely Marvin .NET version 5.4.1.1062 and saved in pdb file format.

## Preparation of protein:

The protein structure was obtained from protein data bank.PDB is a worldwide archive of structural data of biological macromolecule. For this present study the different target protein discussed above was downloaded from RCSB PDB database. The x-ray diffraction structure of the different target proteins under study having resolution not less than 2Å were used for the study. With E.coli as an expression system for docking and downloaded in PDB format. From the above protein structures the heteroatom were removed and their active sites determined using PDB sum database in which liplot the active site of ligand molecule were noted for docking procedure [37-39].

## Prediction of active binding sites:

The most noteworthy step in molecular docking is to locate the ligand-binding sites on the target protein. The protein-ligand binding sites are located using the novel energy-based method known as Q-Site Finder developed by Jackson [40].

## Docking process:

The target proteins after treatment were docked with the ligand lupeol. Molecular docking technique has two molecules that gives a virtually screen on a database of a compounds and help to predict the strongest binders based on their docking score.

## Preparing pdbqt format for ligand and target:

By using AutoDock 4.2 version software, the pdbqt (S) files of protein and the ligand are prepared using AutoDock tool software downloaded from MGL tools [41].

## Procedure:

Grid parameters were generated by altering the dimension of X,Y, and Z to 60. Gpf and dpf file were created to run the autogrid and autodock application with the help of glg files. In genetic algorithms, 25 runs were made to get the desired docking conformation. After running the autogrid and autodock, the analysis procedure were carried out to obtain the docking score, inhibition constant and ligand efficiency value based on their interaction between the protein and ligand molecule with help of conformation procedure and root mean square deviation (RMSD) as table in dlg file [42-45].

## Results:

The present work exhibits the good binding with the proteins used for study namely caspase3, BCL-2, topoisomerase, PTK, mTOR, PI3K, H-Ras and AKT. Among 8 proteins mTOR, Topoisomerase, BCL-2, PTK, H-Ras (Table 1 - see PDF), they showed higher inhibition constant. The protein which are docked with lupeol are mTOR (Figure 1A - see PDF), topoisomerase (Figure 1B - see PDF), Bcl-2 (Figure 1C - see PDF), pkt (Figure 1D - see PDF), PI3K (Figure 1E - see PDF) were found to showed best docking scoring as good binding energy of -11.56kcal/mole,- 7.51 kcal/mole,-6.86 kcal/mole, -6.82 kcal/mole and -7.91 kcal/mole, and has inhibition constant of 6.56 µm,3.12 µm,9.43 µm,10.05 µm and 1.59µm respectively (Table 1 - see PDF).

## Discussion:

Computational methods are useful in making decisions and mimic virtually every aspect of drug discovery and development [46]. For example, in the hit identification phase in which drug discovery teams are provided with many novel chemicals to test for several potential lead molecules that possess the desired drug properties, in-silico method of drug discovery would be an ideal method to use [47].

In this study anticancer activity of lupeol are studied applying molecular docking studies. The ligand-protein docking study done between the different receptor proteins and the ligand Lupeol was presented in Table 1 (see PDF). The scoring function was used to give a good approximation of the binding free energy between a ligand and a receptor, which was usually a function of different energy terms based on a force-field. All ligands docking pose were analyzed, the inhibiting efficiency of the ligand in the process were studied. The strength of the inhibitors interaction between the ligand-protein complexes is shown in Table 1 (see PDF). The ligand binding sites predicted and the comprising the amino acids in the binding pockets are shown in Table 1 (see PDF).

From the docking result, it was identified that lupeol exhibited good binding on Caspase-3 protein and recorded a good binding energy of -11.56kcal/mol and inhibition constant of 6.56 µm and has ligand efficiency of -0.24.Docking of lupeol with mTOR (Figure 1A - see PDF) showed the best binding affinity with binding energy of -11.56 kcal/mol and an inhibition efficiency of 6.56 and a ligand efficiency of -0.22. mTORC1 is activated by PI3k/AKT pathway inhibition of mTOR causes protein translation leads to increased cell growth and proliferation and also in metabolism.

On docking the cancer target enzyme protein Topoisomerase with lupeol (Figure 1B - see PDF) the binding energy was found to be -7.51 kcal/mole and inhibition constant of 3.12 µm and a binding efficiency of 0.24. Topoisomerase involved in various pathological conditions such as mutagenesis a precursor for tumorogenesis.

Docking score of lupeol with BCL-2 (Figure 1C - see PDF) was found to be -6.86kcal/mole indicating good binding and an inhibition constant of 9.43 µm with best ligand efficiency of 0.22µm. From this it showed that B-cell lymphoma (Bcl-2) family of homologue proteins BAX, the primary arbiters of mitochondrial mediated apoptosis. BCL-2 has multiple cell proliferation disorders, other immunodeficiency and infertility. Lupeol with PTK (Figure1D) showed good binding energy, inhibition constant and ligand efficiency of -6.82kcal/mole, 10.05 µm and -0.22 respectively. This PTK have higher inhibition constant. Tyrosine kinase is involved in various functions such as cell death, mutation, carcinoma, and non-hodkins lymphoma. Activation of this pathway leads to the activation of other signalling proteins. When H-Ras and PI3k protein were docked with lupeol (Figure 1E and 1F - see PDF), they exhibited good binding with same binding energy of -7.91kcal/mole, an inhibition constant and ligand efficiency of, 1.59µm and -0.26 respectively. H-Ras and PI3k proteins played major role in lymphoma pathogenesis, GTP- bound forms of Ras protein and their function are cell proliferation, differentiation and apoptosis. PI3k is activated by over expression of AKT pathway which is implicated in cancer, because of it causes growth factor inflammation and DNA damage. Docking of lupeol with AKT and caspase-3 proteins having good binding energy of -8.61 kcal/mole and -8.29 kcal/mole respectively, but it has very less inhibition constant. From our study, it is concluded that lupeol when docked with mTOR, Topoisomerase, BCL-2, PTK, H-Ras and PI3k showed good binding energy and inhibition constant. Further more data were required to elucidate the mechanism of action of lupeol against cancer. Also therapeutic targeting of these proteins such as mTOR, Topoisomerase, BCL-2, PTK, H-Ras and PI3k by lupeol have a long way in alleviating disease caused by deregulation of PI3k, mTOR and PTK pathway such as diabetes mellitus, cardiovascular disorder, autoimmune disorders and neurodegenerative diseases. Therefore lupeol may exert good anti- cancer activity by modulating the PI3k, mTOR and PTK signalling pathway.

## Conclusion:

It is of interest to document the molecular docking analysis data of lupeol with different cancer targets such as Caspase- 3, BCL-2, Topoisomerase, PTK, mTOR, H-Ras, PI3K, AKT. In-silico docking studies using software’s revealed that the lupeol have good docking score minimum inhibition concentration and best affinity towards targeted proteins. Among these eight cancer targets, BCL-2, Topoisomerase, PTK, mTOR and PI3K docking studies showed the best binding energy inhibition constant and ligand efficiency. Thus, it concludes lupeol is one of the significant anticancer phytodrugs.
